# Comparative Transcriptional Analysis of Pulmonary Arterial Hypertension Associated With Three Different Diseases

**DOI:** 10.3389/fcell.2021.672159

**Published:** 2021-07-15

**Authors:** Wei Wang, Zhenhong Jiang, Dandan Zhang, Linghua Fu, Rong Wan, Kui Hong

**Affiliations:** ^1^Department of Cardiovascular Medicine, The Second Affiliated Hospital of Nanchang University, Nanchang, China; ^2^Jiangxi Key Laboratory of Molecular Medicine, The Second Affiliated Hospital of Nanchang University, Nanchang, China

**Keywords:** pulmonary arterial hypertension, gene and pathway enrichment analyses, protein-protein interaction, cell cycle, DNA damage, CUL2

## Abstract

Pulmonary arterial hypertension (PAH) is a severe cardiovascular disorder with high mortality. Multiple clinical diseases can induce PAH, but the underlying molecular mechanisms shared in PAHs associated with different diseases remain unclear. The aim of this study is to explore the key candidate genes and pathways in PAH associated with congenital heart disease (CHD-PAH), PAH associated with connective tissue disease (CTD-PAH), and idiopathic PAH (IPAH). We performed differential expression analysis based on a public microarray dataset GSE113439 and identified 1,442 differentially expressed genes, of which 80.3% were upregulated. Subsequently, both pathway enrichment analysis and protein–protein interaction network analysis revealed that the “Cell cycle” and “DNA damage” processes were significantly enriched in PAH. The expression of seven upregulated candidate genes (*EIF2AK2*, *TOPBP1*, *CDC5L*, *DHX15*, and *CUL1*–*3*) and three downregulated candidate genes (*DLL4*, *EGFL7*, and *ACE*) were validated by qRT-PCR. Furthermore, cell cycle-related genes *Cul1* and *Cul2* were identified in pulmonary arterial endothelial cells (PAECs) *in vitro.* The result revealed an increased expression of *Cul2* in PAECs after hypoxic treatment. Silencing *Cul2* could inhibit overproliferation and migration of PAECs in hypoxia. Taken together, according to bioinformatic analyses, our work revealed that “Cell cycle” and “DNA damage” process-related genes and pathways were significantly dysregulated expressed in PAHs associated with three different diseases. This commonality in molecular discovery might broaden the genetic perspective and understanding of PAH. Besides, silencing *Cul2* showed a protective effect in PAECs in hypoxia. The results may provide new treatment targets in multiple diseases induced by PAH.

## Introduction

Pulmonary arterial hypertension (PAH) is characterized as a cardiovascular disorder associated with multiple clinical diseases that leads to a progressive increase in pulmonary vascular resistance and eventually induces right heart failure. According to the guideline, PAH is classified into seven groups due to different etiologies (Galie et al., 2016). In the various related diseases, it is already known that mutations of heritable pathogenic genes, like *BMPR2*, can result in the suspicious diagnosis of particular PAH, such as idiopathic or heritable PAH (Galie et al., 2016). Besides the well-recognized heritable genetic component, DNA damage (Meloche et al., 2014), microRNAs (Caruso et al., 2017), cellular metabolism (Li et al., 2016), and mitochondrial function (Archer et al., 2010) also contribute to the development of PAH. These molecular mechanism changes can result in the same histopathology in PAH. The following can be observed: increased inflammation; irregulated metabolism or overproliferation of pulmonary arterial endothelial cells (PAECs); and transformed phenotypes, overproliferation, and apoptosis resistance of pulmonary arterial smooth muscle cells (PASMCs) in pulmonary vessels. Although patients can benefit from treatment targeting vasoconstriction, the mortality rate of PAH is approximately 2.8–21.2% 1 year after diagnosis (Hoeper et al., 2017). Until now, the joint mechanism of PAHs associated with different diseases has not yet been systematically analyzed.

The rapid development of high-throughput “omics” technologies (such as DNA microarrays and next-generation sequencing) has provided an increased opportunity to employ computational systems biology approaches to analyze PAH. In 2004, a microarray-based analysis revealed that the expression pattern of 106 differentially expressed gene (DEG) sets could discriminate between PAH patients and normal patients with high accuracy (Bull et al., 2004). Then, a machine learning-based microarray analysis showed that low-expression genes could also be extremely informative at predicting and distinguishing among different forms of PAH (Cui et al., 2019). Recently, a transcriptomic analysis of PASMCs revealed that genes related to cell proliferation and mitosis are increased in PAH (Luo et al., 2020). However, to date, most studies have been focused on PAHs associated with single disease (Desai et al., 2012). Analyzing the molecular commonality of PAHs associated with different diseases will help us better understand PAH.

In the present study, we comparatively analyzed gene expression pattern among CHD-PAH, CTD-PAH, and IPAH. Based on transcriptional dataset GSE113439, we identified 1,442 consistently differentially expressed genes (CDEGs) in PAHs associated with the above three different diseases. Of which, several genes were the known genes related to PAHs such as *ATP13A3, HEY1*, and *SOX17*, and some genes were candidate genes for further experimental verification, such as *CUL2*, *EIF2AK2*, and *TOPBP1*. Pathway and protein–protein interaction (PPI) network analyses suggested that the “cell cycle” and “DNA damage” were common processes in PAHs associated with different diseases. Subsequently, we validated the mRNA expressions of seven upregulated candidate genes (*EIF2AK2*, *TOPBP1*, *CDC5L*, *DHX15*, *CUL1*, *CUL2*, and *CUL3*) and three downregulated candidate genes (*DLL4*, *EGFL7*, and *ACE*) by qRT-PCR. In-depth analysis revealed that CUL2 protein, a structural protein of E3 ubiquitin ligase, was upregulated in hypoxic-exposed endothelial cells, and the loss of *Cul2* could ameliorate hypoxia-induced endothelial injury *in vitro*. This conclusion provides a new way to understand the commonality of the molecular mechanisms in PAHs and reveals a potential connection between ubiquitination and PAH.

## Article Types

### Materials and Methods

#### Data Collection and Pre-processing

Normalized and log-transformed gene expression data were downloaded from the GEO database^1^. GSE113439 includes 11 healthy control samples, six IPAH samples, four CTD samples, and four CHD samples. Kyoto Encyclopedia of Genes and Genomes (KEGG)^2^ pathway information was downloaded from the Molecular Signatures Database (MSigDB)^3^ (Liberzon et al., 2011). After excluding pathways that were too large (>300 genes) or too small (<5 genes) and removing disease- and drug-related pathways, 146 pathways were kept for further analysis.

#### Differentially Expression Analysis

DEGs between PAH samples and healthy control samples were detected using *Limma* (Version 3.40.6), an R package in Bioconductor that identifies DEGs for RNA-Seq or microarrays and provides an integrated solution for performing differential expression analysis (Ritchie et al., 2015). A Benjamini–Hochberg (BH) corrected *p*-value less than 0.01 and an absolute value of fold change (FC) larger than 1.5 were chosen as the cutoff criteria for DEGs.

#### Gene Ontology Enrichment Analysis

Enrichment analysis of the DEGs for the Biological Processes (BP), Cell Components (CC), Molecular Functions (MF), and gene ontology (GO)^4^ terms was performed using BiNGO (Version 3.0.3) (Maere et al., 2005), a plugin in Cytoscape (Version 3.6.0). BiNGO is a Java-based tool that assesses which GO categories are statistically over- or underrepresented in a set of genes or a subgraph of a biological network. Using the whole annotation of human genes as the reference set, GO terms with BH adjusted *p*-values less than 0.05 were extracted as significantly enriched.

#### KEGG Pathway Enrichment Analysis

To select significant KEGG pathways in each disease, hypergeometric enrichment analysis was performed on individual pathway gene sets. Briefly, DEGs from three diseases were first mapped to each pathway. Then, a hypergeometric test was used to test the enrichment of the DEGs in each pathway and obtain a *p*-value per pathway per disease. Next, Fisher’s combined probability test was used to combine the *p*-values per pathway across the three diseases. Finally, the significantly dysregulated pathways across three diseases were selected as pathways with a BH-corrected combined *p*-value less than 0.05.

#### PPI Network Construction and Analysis

The PPI network was constructed using information obtained from the Retrieval of Interacting Genes (STRING) Database^5^. STRING is an online database resource search tool that is used for the retrieval of interacting genes, including physical and functional associations (Szklarczyk et al., 2019). Only PPIs between CDEGs with a confidence score larger than 700 were selected. The resultant network was visualized and analyzed in Cytoscape. The MCODE (Version 1.5.1) plugin of Cytoscape was used to detect network modules from the resultant network. The parameters for MCODE were degree cutoff = 2, haircut = true, node score cutoff = 0.2, *k*-score = 2, and maximum depth = 100.

#### Primary Cell Isolation and Treatments

Pulmonary arterial endothelial cells were isolated from one male Sprague–Dawley rat (weighing 247.1 g, 6–8 weeks) supplied by the Experimental Animal Centre of Nanchang University and cultured according to the previous protocol (King et al., 2004). All experiments and procedures were carried out following the Guide for the Care and Use of Laboratory Animals (National Institutes of Health Publication, revised 1996). PAECs in five to seven passages were used and identified by typical endothelial cell morphology and positive for VIII-related antigen and CD31 antigen by immunofluorescence staining.

Pulmonary arterial endothelial cells were exposed to hypoxia (N_2_/O_2_/CO_2_ at a 94:1:5 ratio) or normoxia (O_2_/CO_2_ at a 21:5 ratio) as the control for 24 h.

#### Construction of Cul2 Interference Adenovirus

The full-length cDNA sequence of rat *Cul2* was retrieved from NCBI database (NCBI reference sequence: NM_001108417.1), and the *Cul2* interference adenovirus (Ad-CUL2-RNAi) was constructed (GeneChem, Shanghai, China). PAECs were cultured in DMEM with 20% fetal bovine serum (FBS, Gibco) for 24 h in six-well plates, then transiently transfected with adenovirus in a serum-free medium and transferred to cell culture medium with 20% FBS for 6 h. PAECs were infected with adenovirus particles carrying si-*Cul2* (Shanghai Genechem Co., Ltd.) at MOI of 100 for 48 h. The negative adenovirus containing the empty vector was utilized as control (si-NC). The transfection efficiency of adenovirus was detected by Western blotting.

#### Quantitative Real-Time PCR Analysis

Total RNA was extracted from PAECs using TRIzol reagent (Invitrogen, New York, NY, United States). The quality and concentration of RNA were determined by Agilent Bioanalyzer 2100 according to the manufacturer’s instructions. The cDNAs were generated by MMLV transcriptase (BioRAD, United States), and quantitative real-time PCR assays were performed as previously described (Nolan et al., 2006). Triplicate PCR amplifications were performed for each sample, and the mRNA levels were normalized to GAPDH. The comparative threshold cycle method (2^–ΔΔ*CT*^) was applied to estimate the relative gene expression of PAECs between hypoxic and control groups. The primer sequences for 10 candidate genes (*EIF2AK2*, *TOPBP1*, *CDC5L*, *DHX15*, *CUL1*–*3*, *DLL4*, *EGFL7*, and *ACE*) are listed in Supplementary Table 1.

#### Western Blotting

Western blotting was performed as previously described (Khoury et al., 2007). Total protein was extracted from cell lysates by homogenization in extraction buffer containing Western and IP lysis buffer (Beyotime, P0013J) supplemented with 1 mM phenylmethanesulfonyl fluoride (PMSF; Beyotime, ST506). The extracted proteins (20 μg) were fractionated by SDS-PAGE (10% polyacrylamide gels, Sigma Inc., United States). PVDF membrane and primary antibodies with proper concentration were co-incubated overnight at 4°C. The following primary antibodies were used: Cullin1 (1:1,000, ab75817, Abcam), Cullin2 (1:1,000, ab166917, Abcam), and Tubulin (1:1,000, 66240-1-Ig, Proteintech). Membranes were washed three times (10 min once) in TBST, followed by a reaction with goat anti-rabbit or goat anti-mouse horseradish peroxidase (HRP)-conjugated secondary antibodies (1:6,000, Transgen) for 2 h at room temperature. Lastly, protein bands were visualized using enhanced chemiluminescence reagents and analyzed by Quantity One analysis software (Bio-Rad).

#### Immunofluorescence Analysis

Immunofluorescence was performed as described previously (Khoury et al., 2007). First, PAECs were fixed in 4% paraformaldehyde (Beyotime) for 30 min at room temperature and blocked with 1% BSA (Solarbio, A8020) in PBS. Then, PAECs were incubated with primary antibodies at 4°C overnight and secondary antibody conjugated to Alexa Fluor 488 goat anti-mouse and Alexa Fluor 546 goat anti-rabbit (1:500, Molecular Probes, Life Technologies Corporation) in PBS at room temperature for 1 h. The following primary antibodies were used: CD31 (1:100, ab24590, Abcam) and FVIII (1:50, ab236284, Abcam).

#### Cell Proliferation Assay

The PACEs from different groups were seeded onto 24-well plates and cultured overnight. Then, the cells continued to grow at 37°C under normoxic or hypoxic conditions, respectively. Briefly, according to the manufacturer’s protocol, PAECs were stained using the BeyoClick^TM^ EdU Cell Proliferation Kit with Alexa Fluor 488 (Beyotime, C0071S). The images were viewed using the Con-focal microscope (Leica, Germany).

#### Wounding–Healing Assay

The wounding-healing assays were conducted as previously described (Yi et al., 2020) when the PAECs were grown to 80% to 90% confluence in the six-well plate. A small linear scratch was created in the confluent monolayer by gently scraping with a sterile cell scraper as per standard methods. Twenty-four hours later, images of the migrated cells were taken by a digital camera (Nikon, Tokyo, Japan), which was connected to the inverted microscope (Nikon, Japan), and analyzed by the image analysis software. The extent of wound healing was determined by the distance traversed by cells migrating into the denuded area. Representative data were cumulative of three independent experiments.

#### Statistical Analysis

All the experiments were repeated three times, and all statistical data were processed by GraphPad prism 8.0 software. Continuous variables were expressed as mean ± SEM, and categorical variables were expressed as percentages. Continuous variables were in accordance with normal distribution, and the comparison between groups was performed by *t*-test. The categorical variables were analyzed by one-way ANOVA and *p* < 0.05 was considered significant.

## Results

### One Thousand Four Hundred Forty-Two Consistently DEGs Were Identified in Three PAH-Related Diseases

**FIGURE 1:**
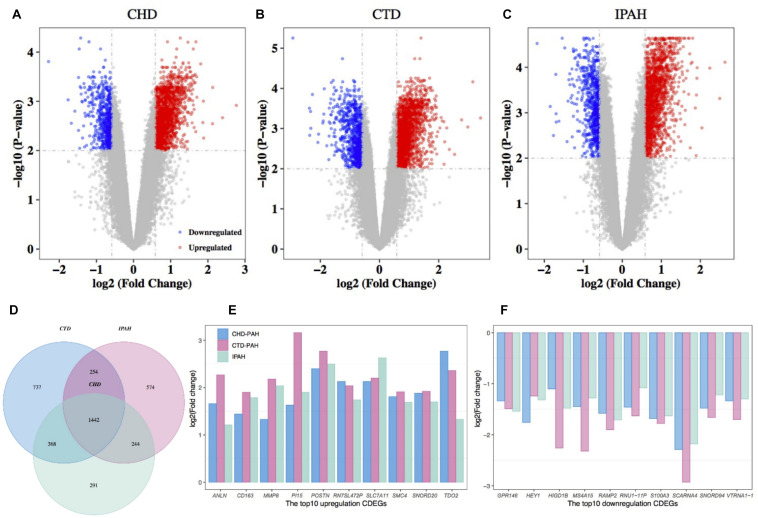
Differential expression analysis of genes in PAH and the corresponding control samples. Volcano plot of the DEGs in CHD-PAH **(A)**, CTD-PAH **(B)**, and IPAH **(C)**. The vertical axis represents the log2-transformed FC and the horizontal axis represents the –log10-transformed BH-corrected *p*-value. The red and blue spots represent upregulated DEGs and downregulated DEGs, respectively, and the gray spots represent non-DEGs. **(D)** Venny plot of the DEGs from the three comparisons. **(E)** The top 10 upregulated CDEGs. **(F)** The top 10 downregulated CDEGs. PAH: pulmonary arterial hypertension.

Differentially expressed gene between the disease samples and their corresponding control samples were inferred using *Limma*. By retaining genes with a BH-corrected *p*-value less than 0.01 and an absolute value of FC larger than 1.5, we obtained 2,345, 2,801, and 2,514 DEGs in CHD-PAH, CTD-PAH, and IPAH, respectively (Figures 1A–C and Supplementary Table 2). By gathering genes that were consistently up- or downregulated in these three diseases, we obtained 1,442 CDEGs (Figure 1D). These CDEGs consisted of 92.7% (1,337) protein coding genes, 4.1% (59) pseudo genes, 2.1% (30) small nucleolar RNAs (snoRNA), and 1.1% (16) other annotated RNAs (Supplementary Table 2). There were 1,158 consistently upregulated genes, accounting for 80.3% of all the CDEGs. The top 10 upregulated CDEGs were *ANLN, CD163*, *MMP8*, *PI15*, *POSTN*, *RN7SL472P*, *SLC7A11*, *SMC4*, *SNORD20*, and *TDO2*. The top 10 downregulated CDEGs were *GPR146*, *HEY1*, *HIGD1B*, *MS4A15*, *RAMP2*, *RNU1-11P*, *S100A3*, *SCARNA4*, *SNORD94*, and *VTRNA1-1* (Figures 1E,F). Among these top CDEGs, there were three snoRNA (namely, *SNORD20*, *SCARNA4*, and *SNORD94*) and one vault RNA (*VTRNA1-1*).

### GO Enrichment Analysis Revealed the Biological Function of the CDEGs

**FIGURE 2:**
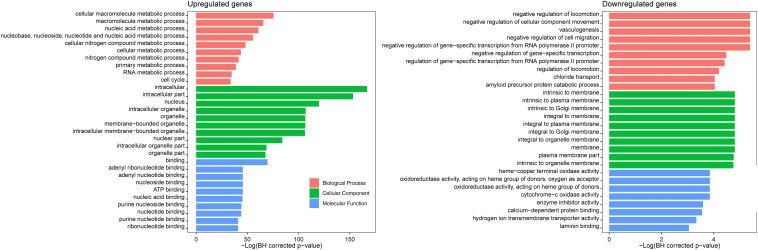
GO enrichment analysis of 1,158 upregulated CDGEs and 284 downregulated CDEGs.

Gene ontology enrichment analysis of the 1,442 CDGEs was performed using BiNGO (Supplementary Table 3). The top 10 enriched GO terms for BP, CC, and MF for the 1,158 upregulated CDGEs and 284 downregulated CDEGs are shown in Figure 2. In particular, the significantly enriched BPs of the upregulated CDEGs included cellular macromolecule metabolic processes (hypergeometric test, BH corrected *p*-value = 1.59 × 10^–33^), nucleic acid metabolic process (BH corrected *p*-value = 3.85 × 10^–27^), and cell cycle process (BH corrected *p*-value = 2.36 × 10^–15^). For the downregulated CDEGs, the enriched BPs included negative regulation of cell migration (BH corrected *p*-value = 4.58 × 10^–3^), vasculogenesis (BH corrected *p*-value = 4.58 × 10^–3^), and negative regulation of cellular component movement (BH corrected *p*-value = 4.58 × 10^–3^). For CC, the intracellular (BH corrected *p*-value = 3.79 × 10^–73^) and membrane (BH corrected *p*-value = 8.17 × 10^–3^) components were the most significantly enriched GO terms in the up- and downregulated CDEGs, respectively. In MF, the most significantly enriched GO terms for upregulated and downregulated CDEGs were “binding” (BH corrected *p*-value = 5.82 × 10^–31^) and cytochrome-c oxidase activity function (BH corrected *p*-value = 2.13 × 10^–2^), respectively. In conclusion, the CDEGs were primarily enriched in metabolic, cell cycle, and binding processes.

### Sixteen KEGG Pathways Were Consistently Dysregulated in Three PAH-Related Diseases

**FIGURE 3:**
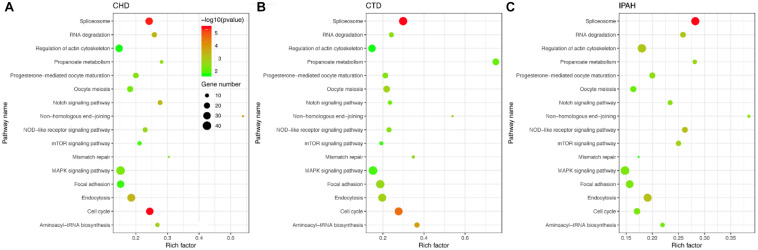
Scatter plot of the enriched KEGG pathways in **(A–C)** CHD-PAH, CTD-PAH, and IPAH. The *y*-axis represents the name of the pathway, and the *x*-axis represents the richness factor, which indicates the ratio of the number of DEGs to the total gene number in a certain pathway. The size and the color of the dots represent the number of DEGs and the range of *p*-values, respectively.

To identify the KEGG signaling pathways, we tested the enrichment of the CDEGs in each pathway. We downloaded KEGG pathway information from MSigDb. Then, hypergeometric enrichment analysis of each pathway helped us obtain a *p*-value per pathway per disease. Finally, by combining the *p*-values per pathway across all three diseases using Fisher’s combined probability test and correcting for multiple comparisons using BH correction, we measured the shared significance of pathways across PAHs associated with three different diseases. By selecting pathways with a BH-corrected *p*-value less than 0.05, we obtained 16 pathways that were dysregulated in PAHs associated with three different diseases (Figure 3 and Table 1). The top three dysregulated pathways were “Spliceosome,” “Cell cycle,” and “Non-homologous end-joining.”

**TABLE 1:** Sixteen dysregulated pathways in PAHs associated with three different diseases.

Standard name	Brief description	*p*-value*
KEGG_SPLICEOSOME	Spliceosome	3.62 × 10^–15^
KEGG_CELL_CYCLE	Cell cycle	5.46 × 10^–9^
KEGG_NON_HOMOLOGOUS_ END_JOINING	Non-homologous end-joining	3.76 × 10^–6^
KEGG_ENDOCYTOSIS	Endocytosis	5.50 × 10^–6^
KEGG_AMINOACYL_TRNA_ BIOSYNTHESIS	Aminoacyl-tRNA biosynthesis	1.54 × 10^–5^
KEGG_RNA_DEGRADATION	RNA degradation	1.82 × 10^–5^
KEGG_NOD_LIKE_RECEPTOR_ SIGNALING_PATHWAY	NOD-like receptor signaling pathway	6.44 × 10^–5^
KEGG_NOTCH_ SIGNALING_PATHWAY	Notch signaling pathway	3.12 × 10^–4^
KEGG_PROGESTERONE_ MEDIATED_ OOCYTE_MATURATION	Progesterone-mediated oocyte maturation	8.55 × 10^–4^
KEGG_PROPANOATE_ METABOLISM	Propanoate metabolism	1.03 × 10^–3^
KEGG_OOCYTE_MEIOSIS	Oocyte meiosis	1.20 × 10^–3^
KEGG_FOCAL_ADHESION	Focal adhesion	1.77 × 10^–3^
KEGG_MTOR_ SIGNALING_PATHWAY	mTOR signaling pathway	3.26 × 10^–3^
KEGG_REGULATION_OF_ ACTIN_CYTOSKELETON	Regulation of actin cytoskeleton	3.26 × 10^–3^
KEGG_MISMATCH_ REPAIR	Mismatch repair	4.63 × 10^–3^
KEGG_MAPK_SIGNALING_ PATHWAY	MAPK signaling pathway	7.92 × 10^–3^

*p-values are corrected for multiple comparisons using Bonferroni correction.

According to their superior pathways, the 16 dysregulated pathways were classified into five groups. The first group included five pathways that were all involved in cellular processes, namely, “Cell cycle,” “Endocytosis,” “Oocyte meiosis,” “Focal adhesion,” and “Regulation of actin cytoskeleton.” The second group was related to genetic information processing and also contained five pathways, namely, “Spliceosome,” “Non-homologous end-joining,” “Aminoacy1-tRNA biosynthesis,” “RNA degradation,” and “Mismatch repair.” The third group consisted of three signaling pathways, namely, “Notch, mTOR, and MAPK signaling pathways,” and their super pathways were signal transductions. The fourth group contained “Progesterone-mediated oocyte maturation” and “NOD-like receptor signaling pathway,” which belonged to the organismal systems. The remaining pathway “Propanoate metabolism” was the fifth group and its super pathway was carbohydrate metabolism. Among the 16 pathways, the Notch, mTOR, and MAPK pathways have already been shown to be related to PAH pathogenesis. Notch1 signaling plays a critical role in PAH by regulating endothelial proliferation and apoptosis (Dabral et al., 2016). The mTOR pathway contributes to the proliferation and survival of IPAH PASMCs *in vivo* (Goncharov et al., 2014). As for the MAPK signaling pathway, PASMCs from PAH display abnormal proliferation as they demonstrate continued growth under non-proliferative, non-growth stimulated conditions, which is dependent on the JNK and MAPK signaling pathway (Wilson et al., 2015). In one word, pathways involved in cellular processes, genetic information processing, and metabolism were tightly connected with PAHs associated with the three diseases.

### PPI Network and Module Analyses Identified the Common High Hubs and Modules

**FIGURE 4:**
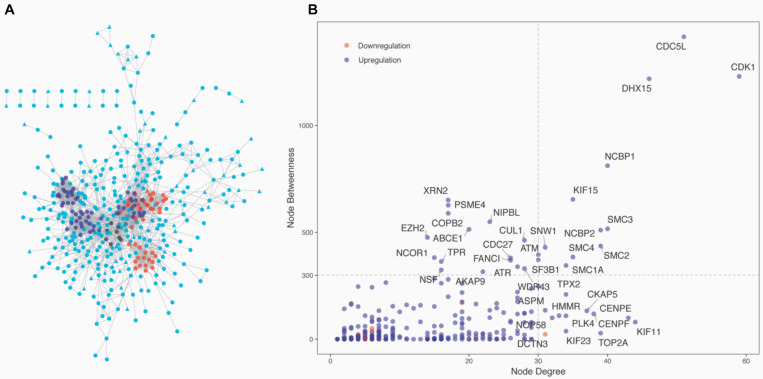
Network analysis of the 1,337 protein-coding CDEGs. **(A)** Overview of the constructed PPI network of the CDEGs. The whole network consisted of 456 CDEGs and 2,337 edges. Upregulated and downregulated CDEGs are represented by square and triangle nodes, respectively. The top three most significant modules are red, blue, and black. **(B)** Scatter plot with the degree (the *x*-axis) and betweenness (the *y*-axis) values for 456 CDEGs. The nodes with the highest values of degree (hubs) or betweenness (bottlenecks) are in the right upper quadrants. The official gene symbol for 26 hubs and 15 bottlenecks are highlighted along with each node.

The 1,337 protein-coding CDEGs were used to construct a PPI network. By selecting PPIs with a confidence score greater than 700 in the STRING Database, we obtained a network consisting of 456 CDEGs (nodes) and 2,337 interactions, accounting for 31.6% of the total CDEGs (Figure 4A). The remaining 986 CDEGs did not fit into the final PPI network. Among the 456 included CDEGs, 408 were upregulated genes and 48 were downregulated genes. Their degree and betweenness values were calculated and visualized in Figure 4B. Nodes with a degree not less than 30 (hubs) or a betweenness not less than 300 (bottlenecks) were highlighted. Finally, 41 genes were screened out (Supplementary Table 4). Among these hubs, 11 genes that shared a high degree and betweenness were defined as “high hubs” (Table 2), namely, *CDK1, CDC5L, DHX15, NCBP1, SMC3, NCBP2, SMC2, SMC4, KIF15, SMC1A*, and *SNW1*. In the list, the cell cycle process-related genes were in majority. Except for one downregulated gene (*DCTN3*), all of the genes were upregulated.

**TABLE 2:** The 11 CDEGs with the highest values of degree and betweenness in the PPI network.

Gene	Full name	Degree	Betweenness	Type
CDK1	Cyclin Dependent Kinase 1	83	2,955.08	High hub
CDC5L	Cell Division Cycle 5 Like	70	4,769.43	High hub
DHX15	DEAH (Asp-Glu-Ala-His) Box Polypeptide 15	46	1,218.76	High hub
NCBP1	Nuclear Cap Binding Protein Subunit 1	40	812	High hub
SMC3	Structural Maintenance of Chromosomes 3	40	516.41	High hub
NCBP2	Nuclear Cap Binding Protein Subunit 2	39	510.38	High hub
SMC2	Structural Maintenance of Chromosomes 2	39	436.39	High hub
SMC4	Structural Maintenance of Chromosomes 4	35	384.46	High hub
KIF15	Kinesin Family Member 15	35	654.24	High hub
SMC1A	Structural Maintenance of Chromosomes 1A	34	345.03	High hub
SNW1	SNW Domain Containing 1	31	429.52	High hub

From the PPI network, we obtained 11 modules with at least five genes (Supplementary Table 4). The top three significant modules are displayed in Figure 4A. Functional enrichment analysis revealed that these modules were mainly related to RNA splicing (BH-corrected *p*-value = 7.47 × 10^–19^), cell cycle (BH-corrected *p*-value = 2.75 × 10^–16^), and DNA repair (BH-corrected *p*-value = 1.51 × 10^–11^). We observed that genes involved in cell cycle and DNA repair were functional together by organizing them into network modules.

### Validation of Selected Candidate Genes Using qRT-PCR in PAECs

**FIGURE 5:**
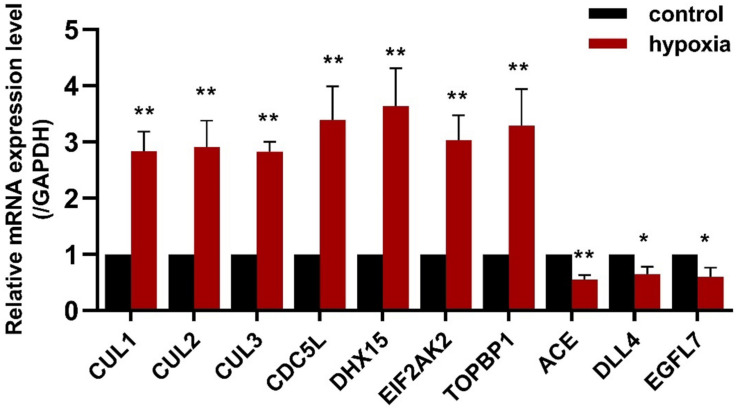
The expression of seven upregulated CDEGs (*CUL1*, *CUL2*, *CUL3*, *CDC5L*, *DHX15*, *EIF2AK2*, and *TOPBP1*) and three downregulated CDEGs (*ACE, DLL4*, and *EGFL7*) was validated using qRT-PCR. All 10 genes were significantly differentially expressed in PAECs under hypoxia for 24 h compared with normoxia control (*n* = 3; *p < 0.05, **p < 0.01).

To validate the results of the gene expression from high-throughput transcriptional data, seven upregulated CDEGs (*EIF2AK2*, *TOPBP1*, *CDC5L*, *DHX15*, *CUL1*, *CUL2*, and *CUL3*) and three downregulated CDEGs (*DLL4*, *EGFL7*, and *ACE*) were selected for qRT-PCR analysis. Of which, *CUL1*, *CDC5L*, and *DHX15* were also hubs with a high degree. qRT-PCR analysis showed that all 10 genes were significantly differentially expressed in the hypoxic group compared with the normoxia group (Figure 5).

### Loss of CUL2 Expression Ameliorates PAEC Proliferation and Migration

**FIGURE 6:**
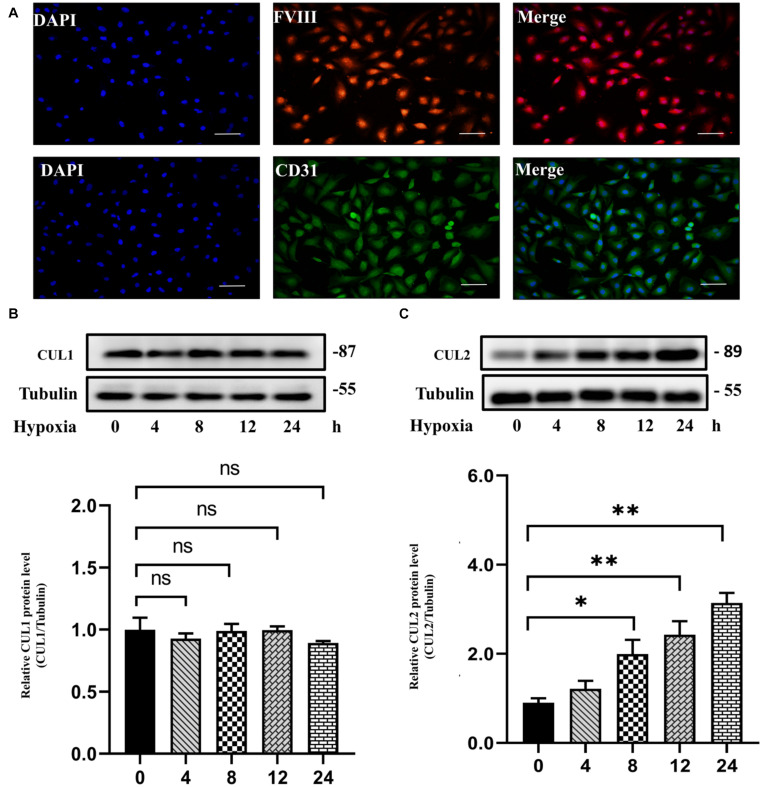
The protein expression of CUL1 and CUL2 under hypoxia for 24 h. **(A)** PAECs were positive for factor VIII-related antigen and CD31 antigen by immunofluorescent staining. **(B)** The protein expression of CUL1 showed no difference under hypoxia for 24 h (*n* = 3; ns: not significant). **(C)** CUL2 protein level was significantly increased since incubated in hypoxia for 8 h until 24 h (*n* = 3; *p < 0.05; **p < 0.01).

**FIGURE 7:**
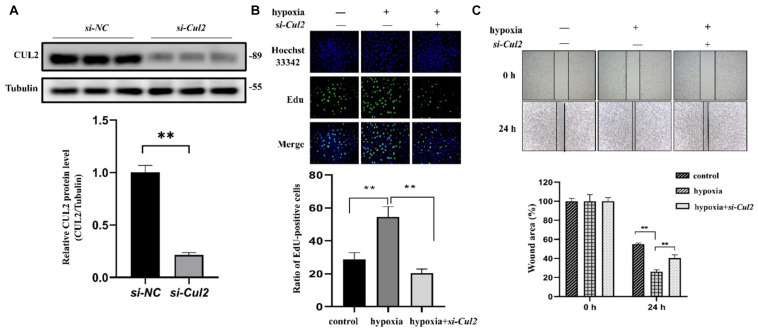
The proliferation and migration of PAECs were decreased after silencing *Cul2***. (A)** The protein expression of CUL2 was significantly decreased in PAECs when interferenced by adenovirus (*n* = 3; **p < 0.01). **(B)** EdU assay to detect the proliferation of PAECs. Cells were divided into control group, hypoxia group, and hypoxia + si-*Cul2* group. Hoechst 33342 was labeled as nuclear staining. The cells labeled EdU were in the proliferative state. Cell proliferation increased under hypoxia and significantly decreased after silencing *Cul2* (magnification: ×200) (*n* = 10; **: *p* < 0.01). **(C)** Wounding–healing assays to detect the migration ability of PAECs. Migration of cells was increased under hypoxia for 24 h and significantly decreased after silencing *Cul2* (magnification: ×200) (*n* = 10; **p < 0.01).

Both GO and pathway enrichment analysis revealed significantly enriched results in the cell cycle process, so we further checked genes involved in the cell cycle process (Supplementary Table 5) from KEGG pathway analysis and found that, *CUL1–3*, encoding the structural proteins of E3 ubiquitin ligase, seem to be attractive. *CUL1–3* can mediate the ubiquitination of proteins involved in cell cycle progression. Bioinformatic analyses showed that *CUL1–3* were all differentially upregulated in PAHs associated with three diseases. qRT-PCR also validated the upregulation of *CUL1–3* in PAECs in hypoxia (Figure 5). Therefore, *CUL1* and *CUL2* were selected for further experimental verification. To confirm the role of *CUL1* and *CUL2*, PAECs were used and confirmed by immunofluorescence assays (Figure 6A). The data demonstrated that the expressions of *CUL1* were not significantly changed (Figure 6B). However, the expression of *CUL2* in hypoxia was significantly increased in a time-dependent manner and maintain at 24 h (Figure 6C). Furthermore, to verify whether *CUL2* was involved in hypoxic-induced endothelial injury, *Cul2* knockdown virus was applied. The efficiency of *Cul2* knockdown is listed in Figure 7A. As expected, the increased proliferation and migration of PAECs induced by hypoxia were both significantly inhibited when silencing *Cul2* gene after 24-h hypoxic treatment (Figures 7A–C). This indicated that loss of *Cul2* expression could ameliorate hypoxic-induced endothelial injury, which is the key feature of PAH.

## Discussion

Pulmonary arterial hypertension is a devastating disease with a poor prognosis and limited therapeutic options. With the rapid development and wide application of high-throughput omics approaches for PAH, large-scale transcriptional data are available from public databases, and multiple studies have been performed to explore the molecular mechanisms of PAH based on these public data (Gharib et al., 2005). Although PAH can be idiopathy or secondary to multiple different diseases, no study has analyzed the molecular commonality of PAHs associated with different diseases. Therefore, in this work, we integrated gene expression data, KEGG pathway information, and PPI networks to analyze the molecular commonality of CHD-PAH, CTD-PAH, and IPAH. Subsequently, an *in vitro* experiment was conducted to elaborate the potential roles of *CUL2* in PAECs after hypoxic treatment. We found that genes and pathways involved in cell cycle and DNA damage are the common molecular mechanisms of CHD-PAH, CTD-PAH, and IPAH. We also proved that gene *CUL2* plays an important role in hypoxic-induced overproliferation and migration in PAECs for the first time.

Based on differential expression analysis, we totally identified 1,442 CDEGs among CHD-PAH, CTD-PAH, and IPAH, with 1,158 upregulated and 284 downregulated CDEGs. Among these genes, our approach revealed some key genes involved in PAH, corresponding to prior studies. The significantly upregulated gene *ATP13A3* (*ATPase 13A3*) was newly found as a pathogenic mutant gene in PAH by whole-genome sequencing (Graf et al., 2018). One of the top 10 downregulated CDEGs, *HEY1*, was found to be significantly altered in PASMCs in growth-restricted rats, and blocking the Notch3-HEY1 signaling pathway in PASMCs could reduce the mean pulmonary arterial pressure (Wang et al., 2019). *SOX17* is also in the CDEG list. It is a new risk gene for CHD-PAH, IPAH, and heritable PAH that impairs the formation of lung microvessels and the function of pulmonary endothelial cells (Graf et al., 2018). In addition to these known CDEGs, we also provided new candidates for further verification, such as *EIF2AK2* (*Eukaryotic Translation Initiation Factor 2 Alpha Kinase 2*) and *TOPBP1* (*Topoisomerase DNA II Binding Protein 1*). *EIF2AK2* is homologous to the known PAH pathogenic gene *EIF2AK4*, a diagnostic marker of pulmonary venous occlusive disease (Eyries et al., 2014) and pulmonary capillary hemangiomatosis (Best et al., 2014). *TOPBP1* is a susceptible gene in IPAH from whole-exome sequencing performed in 2014, and its expression is reduced in PAECs of IPAH (de Jesus Perez et al., 2014). However, another exome sequencing study indicated that *TOPBP1* is unlikely to be the monogenic cause of PAH pathogenesis based on its allele frequency in the background population and prediction analysis (Abbasi et al., 2018).

Besides, there were also 3.2% non-coding RNA among the CDEGs, like snoRNA and lncRNA (Supplementary Table 2). snoRNAs are a group of molecules that range between 60 and 300 nucleotides in length and are involved in the regulation of posttranscriptional modification of ribosomal RNAs. Although the role of snoRNA in the progression of PAH is unclear, 30 differentially expressed snoRNAs were identified in this work. The roles of snoRNAs in lung cancer have been widely investigated (Mourksi et al., 2020). SNORA7B (Cui et al., 2021) and SNORA78 (Zheng et al., 2015) function as promoters in the tumorigenesis of non-small cell lung cancer. lncRNAs are a type of ncRNAs that exceed 200 nucleotides and exert multiple regulatory functions. Three differentially expressed lncRNAs listed in the CDEGs, namely, ILF3-AS1 (Chen et al., 2021), JPX (Li et al., 2021), and RPPH1 (Wu et al., 2020), have already been reported to be involved in the progression of lung cancer by affecting cell proliferation and migration. These dysregulated genes are worthy of further study to find their roles in PAH.

We observed that the KEGG pathway “cell cycle” was one of the top three dysregulated pathways. Investigating the cell cycle pathway, genes from *SMC* (*Structural Maintenance Of Chromosomes*), *CUL* (*Cullin*), *CDK* (*cyclin-dependent kinase*), and the *Cyclin* families were outstanding. Downregulated expression of *SMC1A*, *SMC3*, and *SMC4* could induce growth suppression in lung cancer cells *via* G1/S cell cycle phase arrest and the apoptosis pathway (Zhang et al., 2013). Another PAH integrative analysis revealed that *SMC2* and *SMC4* were high hubs and verified their increased expression in PAH patients compared to control patients (Luo et al., 2020). The *SMCs* family plays key roles in the mitotic cell division machinery (Dávalos et al., 2012), indicating that *SMCs* might be regulators of cell proliferation. *CDKs* and *Cyclins* always work as complexes in modulating progression (Martínez-Alonso and Malumbres, 2020). Regulation of CDK activity at the G1/S phase is important for modulating the organization of DNA replication (Singh and Wu, 2019). Members of *Cyclin* family also act as regulators to ensure DNA replication and chromosome segregation (Martínez-Alonso and Malumbres, 2020). Under hypoxic conditions, increased expression of Cyclin E and Cyclin A could increase more cells from G/G phase to S phase in PASMCs (Xu et al., 2019). In conclusion, genes and pathways related to the “cell cycle” can adjust PAH by regulating the proliferation of PASMCs in the pulmonary vasculature.

Except for these highly DEGs and pathways, high hubs in the PPI network are also directly related to the cell cycle process, such as *CDC5L* and *DHX15*. *CDC5L* is a key regulator of mitotic progression and is critical for maintaining normal proliferation and apoptosis of PASMCs (Crosswhite and Sun, 2014). *DHX15* is an ATP-dependent RNA helicase that has been shown to influence the risk of emphysema in chronic obstructive pulmonary diseases (Manichaikul et al., 2014). Besides, our work also found that genes involved in DNA repair were grouped into a network module. In human PAH arteries and PASMCs, increased DNA damage markers were accompanied by overexpression of DNA repair enzymes (Hu et al., 2013). Inhibition of DNA repair could reverse pulmonary arterial pressure and right ventricular hypertrophy *in vivo* (Hu et al., 2013). DNA damage can act as a trigger of the pathogenesis of PAH (Ranchoux et al., 2016).

In order to assess the reliability of candidate genes (*EIF2AK2, TOPBP1, CDC5L, DHX15*, and *CUL1–3, DLL4*, *EGFL7*, and *ACE*), their expression was validated by qRT-PCR. All 10 candidate genes were differentially expressed under hypoxic conditions, which confirmed the bioinformatic results. As the CUL gene family can mediate the ubiquitination of proteins in cell cycle progression and the importance of cell cycle in PAH, *CUL1–2* were selected for further verification. Experiments were performed to verify the protein expression of CUL1 and CUL2 in PAECs using Western blotting. We found that the protein expression of CUL2 was significantly increased in hypoxia. Moreover, silencing *Cul2* could inhibit the overproliferation and migration of PAECs in hypoxia. CUL2 could mediate the degradation of its ubiquitinated substrate HIF-1α under normal oxygen conditions. Molecular mechanism analysis showed that HIF-1α cannot be ubiquitinated during hypoxia, which resulted in the accumulation of HIF-1α (Kapitsinou and Haase, 2008). Accumulated HIF-1α triggered pulmonary arterial remodeling by regulating its target genes iNOS, VEGF, and heme oxygenase. The results we obtained from the experiment suggested that decreased *Cul2* expression may inhibit the degradation of HIF-1α to develop PAH in hypoxia.

There are several limitations to this study. First, we only gathered data from three types of PAHs, namely, CHD-PAH, CTD-PAH, and IPAH. In fact, PAH is associated with many different diseases. With the rapid development of technologies and their application in pulmonary hypertension, more transcriptional data will be deposited into public databases. Comparative analyses of pulmonary hypertension from more groups will allow more robust results. Second, the screened gene *Cul2* was simply found to be related to PAH based on a microarray *in vitro* or *in vivo* verification. Future molecular biological experiments are needed to verify whether *Cul2* inhibits cell proliferation and migration *via* interfering the HIF-1α ubiquitinational degradation.

## Conclusion

In summary, we performed a comparative analysis of the molecular commonalities of PAHs associated with three different diseases by integrating transcriptional data and pathway information. We identified 1,442 CDEGs, of which 1,158 genes were upregulated. GO and KEGG pathway analyses revealed that the cell cycle and DNA damage processes were significantly enriched. Additionally, hubs and modules from PPI network analysis were also associated with cell cycle and DNA damage processes. Subsequently, the expression of 10 candidate genes related to PAH were validated using qRT-PCR, namely, *CUL1, CUL2, CUL3, CDC5L, DHX15, EIF2AK2*, *TOPBP1, DLL4*, *EGFL7*, and *ACE.* Further cell experiment showed that the *Cul2* expression was increased in PAECs under hypoxia. Silencing *Cul2* could inhibit the overproliferation and migration of PAECs in hypoxia. Therefore, the cell cycle and DNA damage processes are deeply important in CHD-PAH, CTD-PAH, and IPAH, and gene *Cul2* could regulate PAECs in hypoxia. Our study provides a new insight into understanding the commonality of the molecular mechanisms in PAHs associated with different diseases, and *Cul2* deserves further verification to confirm the relationship between ubiquitination and PAH.

## Data Availability Statement

Publicly available datasets were analyzed in this study. This data can be found here: GEO database (https://www.ncbi.nlm.nih.gov/geo/); Accession number GSE113439.

## Ethics Statement

The animal study was reviewed and approved by The Animal Ethics and Experimentation 300 Committee of Nanchang University.

## Author Contributions

WW and ZJ performed the computational analysis of the entire project. DZ performed the experimental test. LF collected the data. WW, ZJ, and DZ drafted the first version of the manuscript. KH was responsible for the entire project and revised the draft of the manuscript. All authors took part in the interpretation of the results and preparation of the final version of the manuscript.

## Conflict of Interest

The authors declare that the research was conducted in the absence of any commercial or financial relationships that could be construed as a potential conflict of interest.

## Footnotes

1 https://www.ncbi.nlm.nih.gov/geo/

2 https://www.kegg.jp/kegg/

3 http://software.broadinstitute.org/gsea/msigdb/

4 http://www.geneontology.org/

5 http://www.string-db.org/

## Supplementary Material

Supplementary Table 1 Primer sequences for ten candidate genes.

Supplementary Table 2 DEGs detected from CHD-PAH, CTD-PAH and IPAH.

Supplementary Table 3 Annotation results for 1442 CDEGs.

Supplementary Table 4 41 Hubs and 11 modules from PPI network.

Supplementary Table 5 DEGs in cell cycle process.
